# From invited to uninvited participation (and back?): rethinking civil society engagement in technology assessment and development

**DOI:** 10.1007/s10202-012-0125-2

**Published:** 2012-11-16

**Authors:** Peter Wehling

**Affiliations:** University of Augsburg, Germany

## Abstract

In recent years, citizens’ and civil society engagement with science and technology has become almost synonymous with participation in institutionally organized formats of participatory technology assessment (pTA) such as consensus conferences or stakeholder dialogues. Contrary to this view, it is argued in the article that beyond these standardized models of “invited” participation, there exist various forms of “uninvited” and independent civil society engagement, which frequently not only have more significant impact but are profoundly democratically legitimate as well. Using the two examples of patient associations and environmental and consumer organizations in the field of nanotechnology, it is illustrated that interest-based civil society interventions do play an important role in the polycentric governance of science and technology. In conclusion, some implications for the activities of TA institutions and the design of novel TA procedures are outlined.

## Abstract (de)

Die Beteiligung von Bürgern und zivilgesellschaftlichen Gruppierungen an Wissenschaft und Technikentwicklung ist in den letzten Jahren fast gleichgesetzt worden mit der Teilnahme an organisierten Formaten der partizipativen Technikfolgenabschätzung (pTA) wie Bürgerkonferenzen und “Stakeholder-Dialogen”. In dem Beitrag wird demgegenüber argumentiert, dass neben diesen standardisierten Modellen “eingeladener” Partizipation vielfältige Formen der “uneingeladenen”, eigenständigen Beteiligung zivilgesellschaftlicher Gruppierungen existieren, die häufig nicht nur wesentlich wirkungsvoller sind, sondern auch hohe demokratische Legitimität aufweisen. Am Beispiel von Patientenvereinigungen sowie von Umwelt- und Verbraucherverbänden im Feld der Nanotechnologie wird gezeigt, dass interessenorientierte zivilgesellschaftliche Einmischung eine wichtige Rolle bei der polyzentrischen Governance von Wissenschaft und Technik spielt. Abschließend werden einige Implikationen für TA-Institutionen und die Neugestaltung von TA-Prozessen zur Diskussion gestellt.

## Résumé

Depuis plusieurs années, l’engagement civil et des citoyens dans la science et la technologie est devenu presque synonyme avec la participation à des formats d’évaluation de la technologie participative (pTA) organisés de manière institutionnelle, tels que les conférences de consensus ou des dialogs des parties prenantes. Au contraire, l’article argumente que, au-delà des ces modèles standardisés de la participation «invitée», il existerait différentes formes d’engagement de la société civile «non-invitées» et indépendantes qui, souvent, ont non seulement un impact plus important mais qui sont aussi profondément démocratiques. Deux exemples d’associations de patients et d’organisations environnementales et de consommateurs dans le domaine de la nanotechnologie illustrent le rôle important que jouent les interventions de la société civile basées sur l’intérêt dans la gouvernance polycentrique de la science et la technologie. En conclusion, certaines implications concernant les activités des institutions d’évaluation de la technologie et la conception de procédures novatrices d’évaluation de la technologie sont présentées.

## Introduction

Since about the 1990s, considerable hopes in terms of the democratization of science and technology politics have been pinned on the dissemination of participatory technology assessment (pTA) procedures. The consensus conference as developed in Denmark, and similar procedures such as citizens’ juries or panels have turned out to be the most important pTA formats, which have been adopted in many countries. Recently, however, dissatisfaction with these forms of citizens’ or civil society participation has grown among TA experts and social science scholars as well as engaged citizens and civil society groups.^1^ This dissatisfaction mainly results, on the one hand, from the regularly very small or even nonexistent political impact of pTA exercises (see for instance Lyons and Whelan 2010), on the other hand from the fact that participatory formats in many cases fail to achieve one of their most important self-proclaimed objectives, namely to bring alternative social rationalities to the fore. Instead, they often tend to reproduce and duplicate the established expert views on the issues at hand (Braun and Schultz 2010; Bogner 2012).

The following considerations start from the assumption that among the main reasons for such shortcomings are, first, the widespread narrowing of public participation to “invited” (Wynne 2007) or “sponsored” (Bucchi and Neresini 2008) forms of deliberation. Actually, participation in institutionally organized pTA formats has in recent years almost become synonymous with citizen and civil society participation as such (Braun and Schultz 2010: 407; Wynne 2007: 103f.). A second reason for the unsatisfactory outcomes of many pTA procedures is to be seen in the predominance on the conceptual level of a misleading understanding of “pure” or “purified” deliberation. In this view, deliberation is conceived of as a task to be performed primarily by hitherto uninformed, disinterested, unorganized, and therefore supposedly “unbiased” individual citizens. Deliberation thus appears to be incompatible with the articulation of particular interests and political views on the respective technologies by engaged citizens or organized groups (Sect. 2). Contrary to this view, I will argue that public participation in debating and shaping scientific and technological issues can be both more effective and legitimate when concerned groups bring in their interests and needs, their knowledges and experiences, and their normative values. To substantiate this claim, I will refer to my current research on civil society participation in the fields of biomedicine and nanotechnologies,^2^ in order to show that organized groups such as patient associations or environmental and consumer protection organizations do successfully and legitimately engage in processes of scientific knowledge production and technology development according to their interests, objectives and values (Sect. 3). This should be reason enough to rethink both the aims and criteria of success of public participation in science and technology. In conclusion, new tasks and objectives of TA institutions will tentatively be outlined (Sect. 4), such as capacity-building for independent and long-term public participation or the design of novel formats such as the “dissensus conference” projected by Hess (2011). Given the fact that such formats would to a certain extent be sponsored and organized, it is important not to “essentialize” the contrast of invited and uninvited participation. What is needed, instead, are novel designs which aim at combining and mutually reinforcing the virtues of both sponsored and spontaneous forms of civil society participation, that is, for instance, transparency on the one hand, more significant scientific and political impact on the other.

## The limitations of invited participation in science and technology assessment

As is well known, efforts to foster public participation in science and technology have undergone a considerable increase in importance in many countries over the last two or three decades. There are a variety of reasons for this but not least it is a political reaction to earlier massive social protests against particular technologies, above all nuclear energy and agri-biotechnology. Most of the currently favoured participation procedures are examples of what Wynne (2007) has termed “invited” and what Bucchi and Neresini (2008) have termed “sponsored” forms of participation. Citizens or civil society actors are *invited,* often by governmental or academic institutions, to participate in evaluating and/or designing certain fields of research and technology such as stem cell research, genetic testing, or nanotechnology. This central and initializing role of a more or less “official” invitation applies to those participation exercises in which individual citizens are engaged as well as to “stakeholder dialogues” and similar procedures to which civil society organizations (CSOs) are invited. In this section, I will point to the limitations of both these types of invited participation.

In formats such as consensus conferences a group of unorganized “laypersons” address a controversial topic of relevance for science and technology policy and develop a supposedly independent position on it at the end of the procedure. Yet, as many scholars, but also CSO representatives, have emphasized in recent years, these models of participation suffer from several serious limitations. Apart from the fact that they regularly are singular short-term events and in most cases fail to have any political or scientific impact, they are also questionable with regard to their background assumptions about effectiveness and democratic legitimacy of participation. As to the *effectiveness* of participation, Maria Powell and Mathilde Colin have pointed to what they term “participatory paradoxes” which can be observed with respect to many sponsored pTA procedures: “The recruitment of unorganized and nonopinionated citizens (usually volunteers) with little background on the scientific issue at hand is puzzling, given that these are the citizens who are least likely to have the energy, capacity, or collective power to engage with scientists and/or make their voices heard on the political level over the short or long term.” (Powell and Colin 2009: 327) Thus, one can reasonably assume that the substantive results of such procedures are not very likely to be innovative and that their impact on the development of science and technology will turn out to be quite limited. In addition, as Hess (2011: 638) has argued, by separating the lay opinion public from “mobilized counterpublics,” that is from interested and engaged civil society (and scientific) groups and networks opposing mainstream science, the lay public is more easily aligned with official publics and their views.

However, as noted above, there also exist participatory procedures such as stakeholder dialogues which expressly address and include collective, organized actors instead of individual citizens. Yet, these procedures do not really transform the dominant understanding of participation, since CSOs such as environmental or consumer organizations regularly are invited as a kind of advocates or lobbyists of a restricted and predefined interest without having a say in debates on the goals and the direction of research and technology development. In addition, as Braun and Schultz (2010: 415) rightly have observed, “publics based on the idea of consensus and education are held in higher regard and ascribed a higher moral authority than those based on the idea of conflict and struggle.” The legitimacy of the participation of organized groups is held to result exclusively from the fact that they narrowly represent “their” specific interests (e.g., warning against environmental or health risks), while any engagement with wider issues such as the benefits of the technology at hand frequently is considered illegitimate. In these procedures, the participation of CSOs therefore can be effective only in a restricted manner, namely to the extent that the latter succeed in giving a voice to the predefined interests they are held to represent. Participation is ineffective, however, because the views and experiences of these groups are not taken into consideration when it comes to more fundamental questions, for instance, those concerning potential alternatives to the respective technology.

The dominant approach to participation in science and technology is, however, equally puzzling with regard to the *democratic legitimacy* of public participation, because it regards precisely those attributes which would *enable* civil society actors to make meaningful contributions—namely independent knowledge, articulated interests, argumentative skills and political or professional involvement—as a *hindrance* to legitimate engagement in participation processes. Underlying this view is a particular conception of “pure” deliberation that must not be “biased” or “distorted” by prior knowledge, individual or group interest, or political involvement. Corresponding to what can be termed the “classic ideal of deliberative democracy” (Mansbridge et al. 2010: 66), the source of legitimacy in this view lies in the fact that participants, by bracketing their self-interests, become both able and willing to acknowledge only the “forceless force of the better argument” (Jürgen Habermas) and thus arrive at defining the common good. As Mansbridge and collaborators summarize, the classic ideal “aims at consensus and the common good. In most formulations it explicitly excludes negotiation and bargained compromise. It excludes self-interest.” (Ibid.)

In recent years, a lot of important criticism of this ideal has been raised in political philosophy and the social sciences. Critics have argued, for instance, that deliberation which follows the “classic ideal” tends to be in itself socially exclusive since it privileges certain styles of supposedly “rational” communication and deprivileges others (see for instance Young 1996); that the classic ideal underestimates the role of power in deliberative procedures (Shapiro 1999; Young 2001) and fails to acknowledge the importance of plurality and conflict in democratic societies (Mouffe 1999); and that it too restrictively rules out self-interests as ostensibly “contaminating” rational deliberation (Mansbridge et al. 2010). Against this background, the classic ideal of deliberation is held to be “insufficient for a polity ideally based on diversity in opinions and interests” (Mansbridge et al. 2010: 66). Nevertheless, and perhaps surprisingly, it still seems to serve as the conceptual and normative background of many pTA exercises, as critics have observed (see Powell and Colin 2009; Braun and Schultz 2010; Hess 2011; Kleinman et al. 2011; Powell et al. 2011).^3^ Frequently, the consequences of this orientation seem rather questionable: “Participants are conceptualised as citizens or laypeople, meaning that they are interpellated as individuals, not as members of an organisation or an interest group. In addition, their main qualification is exactly their ignorance concerning the issue at stake and, at the same time, their amenability to education.” (Braun and Schultz 2010: 409) In practice, this may result in the exclusion of citizens or groups who are organized and/or already have their own (political) opinions on the issues at stake. Indeed, the participants are expected to enter the procedure as “blank slates” (Kleinman et al. 2011) or “blank minds” (Braun and Schultz 2010); thus, one main purpose of such deliberative formats is educational and can be seen in demonstrating “the capacity of ordinary citizens to refine their views and attitudes through the process” (Braun and Schultz 2010: 410; see also Bogner 2012: 511f.). One should, of course, not underrate this goal; yet in this way, deliberative legitimacy is constructed at the expense of the effectiveness and political or scientific impact of public participation. It is unsurprising therefore that such participatory exercises regularly raise suspicions of serving merely symbolic functions and aiming at securing the social acceptance of contested scientific and technological innovations. In addition, to the extent that the public is constructed as one which is to be educated with regard to the scientific issues at hand, such forms of participation still adhere to a central assumption of the “deficit model” of the public understanding of science.

Deliberative pTA exercises strive to create procedural legitimacy by foreclosing preexisting “biased” views in order to ensure that the result of deliberation is exclusively based on undistorted communication and “the better argument.” Nevertheless, this legitimacy remains vulnerable to being disrupted by adverse influences and occurrences during the participatory procedure. In a recent paper, Alexander Bogner mentions three such adverse developments and emphasizes that the reasons for these “are to be found in the structure of the procedure rather than in any shortcomings on the part of the actors (e.g., poor moderation or incompetent laypeople)” (Bogner 2012: 516). Bogner describes the first of these developments as the “selective nature of deliberation norms;” this means that in the process specific “local” norms of deliberation become established which lead to the exclusion or self-exclusion of those participants “who cannot or do not want to fit in with those norms” (Bogner 2012: 517). The second disruptive factor lies in the fact that the participants tend to increasingly adopt the dominant scientific or ethical framings of the issues at hand, whereas the independent views they had expressed at the beginning of the process are subsequently dropped (ibid.: 517–19). Thirdly, the quality, rationality, and legitimacy of the deliberative process are seriously called into question by what Bogner (ibid.: 519) terms the “primacy of organization” (see on this also Görsdorf 2012). This means that organizational aspects, such as the need to comply with the time schedule of the procedure or to produce a “presentable” final report, factually become the main concerns of both the organizers and the citizens while substantive issues threaten to be marginalized due to time pressure. Obviously, all these factors are likely to question or even undermine the claim to procedural legitimacy of pTA exercises which, according to the “classic ideal,” is held to result from the argumentative quality of unbiased deliberation. To sum up, both the political or scientific effectiveness and the democratic legitimacy of those participatory exercises which draw upon the classic ideal of deliberation and interpellate unorganized individual citizens are contestable—and are, indeed, increasingly contested in current debates.

## The effectiveness and legitimacy of “uninvited” participation

During the last decades, more or less simultaneously to the diffusion of sponsored pTA procedures, one can observe the emergence and intensification of multiple forms of organized and collective civil society participation in science and technology which are to be termed “uninvited” (Wynne 2007), “spontaneous” (Bucchi and Neresini 2008) or “bottom-up” (Powell and Colin 2009). One of the most striking examples of this is patient organizations in the field of rare diseases who are initiating and promoting medical research into reliable diagnostics and promising therapies for “their” respective diseases or are even themselves become research organizations (Terry et al. 2007; Callon and Rabeharisoa 2008; Kanellopoulou 2009; Panofsky 2011).^4^ To achieve these goals, they adopt a wide range of tools and strategies including, for instance, the collection and systematization of experiential knowledge about diseases and therapies, the funding of (usually small) research projects and grants, the announcement of research awards, support for doctoral students, or the creation of collaborative networks among researchers from different institutions and/or countries. In addition, social health movements are calling for “official” medical-scientific recognition and adequate treatments for contested conditions and illnesses (e.g., Gulf War syndrome, multiple chemical sensitivity, fibromyalgia). Other groups criticize what they see as a one-sided orientation of biomedical cancer research to genetic factors and actively engage in the search for environmental causes (air and water pollution, contaminants in food, etc.) of cancers, particularly breast cancer. Environmental and consumer organizations argue for more research devoted to the environmental or health risks of nanotechnology or do initiate and even conduct such research themselves (Hess 2009, 2010). Although their resources are quite limited, they nevertheless contributed to putting the environmental and health implications of nanotechnologies on the “official” research agenda. Finally, we should not forget the advocacy of civil society groups *for* the development of certain technologies (Hess 2007); the support of Greenpeace for the CFC-free refrigerator “Greenfreeze” during the 1990s acquired particular prominence in this regard. Likewise, a significant number of patient associations are coming out in support of specific directions of research and medical technology development, some of them being socially contested such as stem cell research and genetic screening.^5^

The particular features of such forms of civil society participation in science and technology become most sharply visible when we focus on what patient associations actually do when they engage in medical research. Then, we recognize the contours of a model of uninvited and activist participation which differs from the deliberative one in several important respects.^6^*First,* patients do not wait for an invitation to participate but simply start to engage in medical research according to their own needs and priorities, and they do so continuously, not only for a couple of weeks or months. *Second,* they consciously organize themselves, based on their experience that only as an organized collective they will ever have the resources, the capacities and the power to gain influence on medical research and research policies. *Thirdly*, they strive to become as knowledgeable and well-informed as ever possible about those scientific issues that appear relevant to them, that is, they do not act as those “blank minds” that are expected to participate in deliberative procedures. *Fourthly,* and perhaps most importantly, they do not bracket and neutralize their particular interests as a precondition of legitimate participation. Quite to the contrary, their central aim is just to set the interests, needs and values of a particular group of patients on the agenda of science and research politics.^7^

Unsurprisingly, such forms of activist civil society participation prove to be considerably more effective than deliberations among unorganized laypeople. The reasons for this are not very hard to find: Civil society groups and organizations have more or less strong interests in the issues at hand, and they possess relevant knowledge, be it that they have become “lay experts” by having acquired scientific or technical expertise, be it that they command independent experiential or local knowledges which might add to or compete with established scientific knowledge. On a general level, we can distinguish three important functions which civil society actors are able to fulfill for science and technology development or research politics:

- First, they participate in diverse and often conflictual ways in research agenda setting, mainly by focusing on what Scott Frickel and collaborators have termed “undone science” (Frickel et al. 2010; see also Hess 2009, 2010), that is, problems and issues that have escaped the attention of mainstream science. This has been the case with rare or “orphan” diseases which had been neglected for a long time by mainstream medical and pharmaceutical research, yet in recent decades, patient associations succeeded in drawing medical, political, and public attention to this class of diseases. Generally speaking, research fails to be conducted, for example, because it is not economically profitable or generates excessive costs, because it is not politically opportune and not supported by powerful actors, or because it does not seem to be scientifically attractive, as for instance when it promises scant reputational rewards or research funds and presents few career opportunities. Civil society organizations are among the most important actors to oppose to such mechanisms of excluding certain issues from scientific interest (Frickel et al. 2010). Nevertheless, in many cases, civil society actors also argue or agitate for *not* researching certain topics, *not* using certain methods, and *not* developing certain technologies, whether because of presumably inacceptable risks (e.g., nuclear energy, agri-biotechnology, nanotechnologies in food), excessive costs (e.g., nuclear fusion energy), or because of ethical and political concerns (e.g., large-scale geo-engineering or human enhancement).
- Second, civil society actors can provide important epistemic or organizational resources for scientific knowledge production. With regard to epistemic issues, it is primarily the experiential or local knowledge of concerned groups that often proves to be relevant for researchers. Patient associations, for instance, regularly possess very detailed and differentiated knowledge about the course of a disease, and the efficacy of therapies or the everyday needs of patients. Often, they collect and systematize this knowledge—what Michel Callon and Vololona Rabeharisoa have termed “research in the wild” (Callon and Rabeharisoa 2003)—and thus can substantively contribute to the production of scientific knowledge. In organizational and interactive respects, patient associations can be supportive when they bring together research teams from different academic institutions or different countries who work on similar topics but nevertheless do not know each other. In addition, they organize contacts between researchers and patients, which is a particularly important issue in the field of rare diseases where there are often only very few patients in a given country.
- Third, civil society groups can act as an epistemic corrective for scientific research based on their local knowledge or patients’ experiential knowledge, for instance, when they criticize the mainstream definitions of certain environmental or health problems as one-sided and insufficient. One of the most prominent examples of such an epistemic challenge is the “Environmental Breast Cancer Movement” in North America that is contesting the dominant biomedical approach to breast cancer etiology and treatment and its focus on genetic and lifestyle factors (Brown 2007; McCormick 2009a, b). Up to now, the vast majority of funds invested in cancer research flow into cost- and technology-intensive basic research, early detection technologies, or expensive therapies. Movement activists and organizations argue instead for more research into possible environmental causes of breast cancer and a shift of biomedical attention from treatment to prevention. Another case in point is research on the potential health and environmental risks of nanosciences and nanotechnologies. In this regard, civil society actors (such as the Canadian ETC Group) did not only point to the fact that risk research is clearly underrepresented in the overall funding of nanotechnologies. More specifically, they argued that for identifying and assessing the risks of nanoscale products (e.g., their toxicity) new conceptual approaches are needed since it is entirely inappropriate “to extrapolate an understanding of nanomaterial toxicity from our experience with the material in bulk form” (Wickson 2012: 224). In addition, given the extremely complex task of anticipating and assessing nanotechnology risks, CSOs oppose the dominant framing of all nanotechnology issues in narrow terms of “risks versus benefits,” with the promised benefits being seldom as rigorously scrutinized as the suspected risks (Miller and Scrinis 2010; Wickson 2012).

Of course, collective civil society actors are not always successful in achieving their goals related to science, technology, and research policies. Yet, as noted above, there are a number of examples where civil society groups had remarkable success, whether in shaping the research agenda, in supporting scientific knowledge production or in challenging established scientific paradigms.^8^ At any rate, one can reasonably assume that uninvited participation of collective and well-informed actors is more likely to have a substantial impact on the development of science and technology than “laboratory participation” (Bogner) of randomly selected individual citizens.

Whereas the effectiveness of uninvited participation thus might appear unproblematic, how can we understand and justify its democratic and scientific legitimacy given the fact that a lot of civil society groups’ interventions are clearly based on particular interests or even self-interests? As is well known, in many variants of democratic theory, especially in theories of deliberative democracy, interest-based politics is seen as close to mere lobbyism and pressure-group politics. It therefore seems to be hardly compatible with processes of deliberation and democratically legitimate politics that usually is held to be oriented around discussing the common good “rather than competing for the promotion of the private good of each” (Young 1996: 121). However, by closer inspection, this argument proves to be less convincing than it might appear at first sight; in what follows I will briefly argue, mainly using patient associations as an empirical illustration, that the expression of self-interest and deliberative discussion do not necessarily contradict each other and that interest-based activism can indeed be a form of legitimate participation in science and technology.

In democratic societies, it is essential that social actors, in particular marginalized or disadvantaged groups, are able and allowed to express their own specific self-interests, in order to make sure that they can speak for themselves and are not misrepresented by others: “Those who know their interests best, namely (in general) those whose interests they are, need to deliberate with others about those interests, come to understand them, express them, and stand up for them.” (Mansbridge et al. 2010: 72)^9^ Thus, instead of bracketing their interests as a starting point for good deliberation, participants can even become more clearly aware of what their self-interests are in the process of deliberation. As Mansbridge and collaborators continue, “(i)ncluding self-interest in deliberative democracy reduces the possibility of exploitation and obfuscation, introduces information that facilitates reasonable solutions and the identification of integrative outcomes, and also motivates vigorous and creative deliberations” (Mansbridge et al. 2010: 72f.).

In this context, it is crucial to distinguish collectively articulated and reflected interests from mere short-term and surface preferences such as consumer choices on markets (Mansbridge et al. 2010: 68, n. 15). This is the reason why *organized* groups are important for the expression of interests; civil society associations frequently (though of course not always) offer a forum where individual preferences and immediate needs can be reflected and balanced, with the aim of expressing collective interests and values (see Fung 2003).^10^ To put it differently, collective interests are neither a “spontaneous” preference nor an objective and static “given” but themselves the results of (more or less open and fair) processes of interaction and discussion within social groups. For this reason, interest-based politics and activism go far beyond a “privatized consumer orientation” (Young 1996: 121) toward politics (or science and technology) which deliberative democrats rightly reject.

In addition, in many cases, there is no clear-cut distinction between particular group interests and the common interest or common good. Beate Kohler-Koch (2011: 11f.) illustrates this with reference, among other things, to patient organizations in which there is an inseparable mixing of the self-interest of a particular segment of the population with the implementation of universal rights such as the right to equal access to the health-care system. The same applies to environmental or consumer protection organizations which on the one hand promote their particular interests, priorities, and values (e.g., what environmental or health risks are selected and addressed, what helps the organization to gain publicity or receive more funding?), while on the other hand, protection of the environment and consumer health can reasonably be understood as lying in the common interest. Moreover, the common good is not necessarily something that lies “beyond” or “above” all individual and group-specific interests but instead is related to or even composed of the various individual goods. This applies, for instance, to health-care where the common good consists of numerous individual goods, namely of the equal access of all suffering individuals to the health-care system including medical research. In such cases, according to Mansbridge et al. (2010: 75), “the presentation of one’s self-interest is in itself a justification, a reason in itself for adopting a particular policy”. Last, but not least, the reflection on and articulation of one’s self-interest can serve a critical function in processes of deliberation and policy-making. Mansbridge and collaborators rightly remark that *without* the clarification and expression of self-interest the “understandings of the common good of the more powerful in the polity” are likely to dominate, “even without ill will or the intent to exercise power” (Mansbridge et al. 2010: 74). Ironically, under certain circumstances, deliberation can even promote disagreement and conflict of interests instead of reducing it. As Ian Shapiro (1999: 31) notes, while “(p)eople with opposed interests are not always aware of just how opposed those interests actually are,” deliberative processes can bring those differences to the surface. However, as Iris Young (2001) has argued, this is not necessarily the case because frequently deliberative processes are inadvertently influenced by hegemonic discourses. “When such discursive systems frame a deliberative process, people may come to an agreement that is nevertheless at least partly conditioned by unjust power relations and for that reason should not be considered a genuinely free consent.” (Young 2001: 685) The hegemony of such discursive framings might in particular prevent people from recognizing that their self-interests are not, or only partly, represented by what is held to be the common interest. Becoming aware of this and expressing one’s own interests is in such situations an important element of democratic politics; according to Young (2001: 687), achieving this goal may require various nondiscursive means.

As the preceding considerations underline, there are a number of good reasons to acknowledge the fact that the expression and promotion of self-interests should not generally be excluded from deliberative discussions and that insisting on one’s own interests is not necessarily illegitimate and disrupting or even “contaminating” such discussions. To the contrary, the articulation of self-interests can even provide a source of legitimacy for public participation in democratic deliberation and decision-making. As noted above, this applies in particular to those cases in which the common interest is composed of the particular individual interests of all participants, or the interests of some social groups have been neglected by more powerful actors, or hegemonic definitions of the common good would go unchallenged as long as certain groups do not become aware of their opposing interests and fail to publicly articulate them.

The legitimacy of interest-based activism becomes even more clear when we look more specifically at civil society participation in *science and technology*. Contrary to idealized notions of scientific or medical progress, science clearly does not merely develop according to a universal and rational logic of disinterested and value-free truth-seeking. By contrast, external interests and influences, primarily from the state and the economy, as well as internal criteria of selection which are often far from rational determine *what* research is done and *how* it is done.^11^ To the extent that dominant paradigms exclude important and pressing social or medical problems from scientific attention, science obviously is in need of inputs from other sources, in particular from civil society that acts in many instances as a counterpart to the powerful influences of governments, business firms and the institutions of mainstream science. With regard to the science-related activities of patient organizations, Rabeharisoa and Callon therefore speak of a “third way” in research policy that rests on the active participation of affected patients and could serve to compensate and rectify the one-sidedness and limitations of both state- and market-driven research funding. According to these authors, it remains to be clarified, “whether this model can be transposed to other fields than health, such as the environment, energy, or food security” (Rabeharisoa and Callon 2002: 64).^12^ At any rate, civil society groups are important or even indispensable actors of what I would like to term the “polycentric governance” of scientific knowledge production. The sources of legitimacy of their engagement are to be found precisely in the fact that the specific needs, experiences, knowledges, and values of particular concerned groups are *collectively expressed* in the scientific and political sphere. Such interventions are not (or at least not primarily) justified by reference to the common good (although they can of course include an alternative idea of the common interest) but by introducing particular and partial interests, thus irritating and potentially transforming both established political debates and the agenda of scientific research. As Young (2001: 687) rightly emphasizes, the goal of activists often is “to rupture a stream of thought, rather than to weave an argument”.

However, even if both the effectiveness and the legitimacy of activist participation in science and technology can be justified in general, this does of course not mean that they are to be taken for granted in any specific case. In a recent paper on the success of patient advocacy groups and health movements, Steven Epstein (2011: 263–8) has listed three serious complications and obstacles to the effectiveness and legitimacy of activist and interest-based participation: The first one is the problem of *representation*, that is, the question of who speaks for the patient and his interests. As already mentioned above, this problem is indeed crucial since both the performance and the legitimacy of activist participation depend on the fact that it is actually the interests of the patients themselves (or, more general, the “ordinary” group members**)** that are set on the research agenda. The second problem addressed by Epstein is that of *expertise* which refers to possibly emerging intra-group divisions and tensions between “lay experts” who have become familiar with scientific knowledge and “ordinary” group members. In addition, while such a “scientization” of an organization or movement tends to facilitate interactions with and acceptance by scientists, it may on the other hand reduce critical distance of the group or its leaders from the dominant scientific paradigms and research priorities. The third problem is that of *incorporation and co*-*optation* by powerful political and scientific institutions or economic actors. While it is often hard to say whether the incorporation of group interests into existing institutional practices “should be counted as victory or defeat” (Epstein 2011: 267), there are at least some undisputed cases where patient groups are funded and instrumentalized by pharmaceutical companies thus seriously jeopardizing the legitimacy of their activities.

However, although these problems should be taken very seriously, they do not generally question the legitimacy of activist participation in science and technology. Instead, they indicate that both the “external” conditions (for instance adequate financial resources that enable patient groups to reject funding by economic actors) and the “internal” reflexivity and responsibility of activist groups are crucial in order to render such forms of participation successful and legitimate. This leads to the seemingly paradoxical question of how institutions of technology assessment could meaningfully relate to or even support uninvited civil society participation in science and technology—and of why they should do so.

## New tasks and objectives for TA institutions?

In recent years, the evaluation and refinement of pTA methods and procedures have been a central focus (or even *the* central focus) of political and social science debates on public participation and democratization of science and technology. However, if we acknowledge that uninvited civil society participation represents a complementary or competing way to make science, technology and research politics more receptive to societal needs and expectations, the question arises of whether and how TA procedures and institutions could contribute to rendering such forms of participation more visible, more successful, and more reflexive. In this concluding chapter, I would like to present two suggestions that, although starting from different angles, both might help to achieve these goals. Both of them factually relativize the opposition of invited and uninvited participation thus making us aware that it would be untenable to essentialize and normatively or politically overrate this distinction.^13^

a) The first suggestion draws on considerations made by a group of US scholars aiming at fostering more meaningful, effective, and sustainable citizen participation in science and technology (see, for instance, Powell and Colin 2008, 2009; Powell and Kleinman 2008; Powell et al. 2011; Kleinman et al. 2011). These authors’ reflections start from a critique of ongoing participatory exercises, particularly in the field of nanotechnology. They argue that exercises such as consensus conferences regularly have no discernable political or other societal impacts, that their goals are seldom clearly articulated, and that they are unlikely to prepare citizens for ‘real-world’ political participation, “which is long term, seldom controlled or facilitated, and often contentious” (Powell and Colin 2008: 129). This even raises suspicions that many procedures “are simply short-term ‘exercises’ that are analyzed for eventual publication in scholarly journals” (ibid.: 128). In addition, by attempting to create “blank slate” citizen panels, the organizers of pTA procedures “risk the exclusion of some of the most interesting (and interested) and dynamic potential participants” (Kleinman et al. 2011: 237).

As a consequence of these critical objections, the authors emphasize that public engagement initiatives should be designed with the aim of “empowering citizens and slowly building their capacities to engage independently,” so that they can have tangible impacts on science and technology development over the long term (ibid.: 130f.). To achieve this goal, Powell and Colin (ibid.: 133–5) propose ten recommendations that are intended to overcome the shortcomings of established participatory exercises. Here, I can only mention three of these, which seem to be most important with regard to the design of participation processes: TA institutions should, first, develop and incorporate new forms of interaction with citizens that go beyond short-term exercises; engagement processes must, second, be as open-ended as possible, that is, they must not be oriented toward a predefined outcome such as a final report of the citizens group.^14^ Thirdly, “citizen engagement projects should include capacity building, incentives, and training for citizens. Citizens do not ‘naturally’ know how to engage with each other, scientists, or policy makers inclusively and democratically or work collectively to have a voice in political processes.” (Ibid.: 134)

In addition to such more conceptual considerations, some of the above-mentioned scholars have supported and facilitated for several years the formation of an independent citizens group engaging with the development of nanotechnologies. The starting point of this effort has been a consensus conference on nanotechnology organized in 2005 at the University of Wisconsin in Madison (USA). Since several citizens who had participated in the conference wanted to engage further, with the support of Maria Powell and Mathilde Colin they formed an independent group, The Citizens Coalition on Nanotechnologies (CCoN), and subsequently organized a number of follow-up projects such as “Nano Cafes” (see for a detailed account Powell and Colin 2009).^15^ Although Powell and Colin evaluate their engagement in this process rather self-critically and do not at all conceal the enormous difficulties they had faced, they are nevertheless confident that their support and encouragement has nurtured “genuine citizen engagement that goes far beyond other engagement exercises” (Powell and Colin 2009: 340). With regard to TA institutions and pTA procedures two conclusions can be drawn from this example: It demonstrates, first, that even invited participatory processes can give an important impulse for further independent and “uninvited” civil society engagement. Conversely, this means that pTA procedures are not necessarily completed when they have come to their “official” end, but may offer opportunities for long-term engagement of citizens which should be supported by TA institutions. It seems almost needless to repeat that the exclusion of the most interested citizens from the sponsored procedure would openly contradict this aim. Second, citizens would possibly have greater benefit from invited participation when the latter is more tightly and consciously related to the local or regional context. This would be more favorable for citizens’ capacity-building and self-organized activities than procedures on the national level, with participants coming from different regions.

b) The second new task for TA institutions and organizers of participatory procedures I would like to outline here consists in designing novel participatory formats which systematically take into consideration that science and technology development are shaped by a plurality of competing and conflicting interests, perspectives, and paradigms. Thus, an important aim of civil society participation should be to go beyond assessing the risks and benefits of an already established trajectory of research and technology development and to look for alternative directions of research which might prove to be fruitful but are marginalized and neglected by mainstream science. Given this background, David Hess has recently suggested two novel participatory models that take into account the contested nature of both scientific research agendas and epistemic approaches to certain issues and problems. With regard to research agenda setting, he proposes to “allocate a portion of public research funding to a competitive funding process that would seek to identify areas of undone science” (Hess 2011: 638). As noted above, the term “undone science” refers to areas of research which are identified by social movements or scientific counterpublics and potentially include issues of broad public interest but receive systematic inattention within the dominant scientific networks. The second proposal offers an alternative approach to public deliberation and participation in science. Instead of holding a consensus conference, Hess argues, one might initiate a *dissensus conference* “to draw attention to and analyze the perspectives of a scientific counterpublic. The object of the conference would not be to produce a report that provides input from a random selection of laypeople into a technical decision but instead to produce a publicized controversy that draws attention to the power–knowledge issues in a given scientific field.” (Hess 2011: 639) Participants in the dissensus conference would be stakeholders such as leaders of dominant and subordinate networks in the relevant scientific fields, potential funders, civil society and social movement representatives, industry representatives, journalists, and regulators.

What is important in both these models is, on the one hand, that the objects of public discussion and deliberation are not merely the presumable consequences (“risks” or “benefits”) of a more or less established strand of scientific research. What is at stake is instead what alternative and possibly promising research topics have been marginalized, what the reasons for this are and what conflicts and disagreements exist between different scientific paradigms and thought-styles in the field. What we see here is a broader understanding of public participation in science, or of the democratization of science, since even the questions of what gets studied (or not), of how and with what aims it is studied, and of what should possibly studied instead come under public scrutiny. On the other hand, both models suggested by Hess would in a way result in a kind of institutionalization of uninvited and activist participation. However, this would not necessarily be tantamount to domestication and cooptation, but could as well improve the visibility and impacts of civil society participation.^16^ In addition, such institutionalization could to a certain extent also result in increasing the transparency and legitimacy of civil society actors’ engagement since the latter are in a way *authorized* to participate (see Brown 2006: 208–10). Yet this apparently raises the difficult question of who is authorized to participate by whom; the only viable, but always provisional and contestable solution to this problem seems to be to make such formats as inclusive as ever possible.

Given the increasing dissatisfaction with the existing forms of invited public participation in science and technology noted in the introduction, a broader and more differentiated understanding of the issue seems crucial that pays much more attention to the role, the impacts and the virtues of uninvited citizen and civil society participation. Correspondingly, TA institutions and practitioners should start to think about novel designs of participatory procedures which one the hand are revoking the one-sided orientation toward the classic ideal of deliberation and on the other hand integrate experiences and impulses from spontaneous civil society engagement. While there is of course no “one best way” to both effective and democratically legitimate public participation in science and technology, it might now be the right moment to rethink this important challenge and move beyond established routines and practices.

## Footnotes

1 See for instance Wynne 2007; Powell and Colin 2009; Braun and Schultz 2010; Lyons and Whelan 2010; Hess 2011; Delgado et al. 2011; Bogner 2012.

2 The research project “Participatory Governance of Science” is conducted by Willy Viehöver and the author at the University of Augsburg and funded by the German Ministry of Education and Research (BMBF) from 2009 to 2013.

3 To be sure, here I am neither criticizing theories of deliberative democracy as such nor do I want to contribute to discussions on democratic theory in general. The following considerations focus instead on the shortcomings of the classic ideal as well on as their consequences for the conception and organization of participatory procedures. According to Mansbridge et al. (2006: 2) one can distinguish three stages of the evolution of deliberative democracy theory: early theory, a period of criticism in the 1990 s, and recent theoretical formulations which give more room to “real-world” social practices, to conflict, power, interests, or emotions. Therefore, I would not argue that deliberative democracy theory is in principle unable to integrate these phenomena (see for instance Dryzek 2000; Kahane et al. 2010; Mansbridge et al. 2010); however, pTA procedures up to now build upon the classic ideal rather than on those more recent theoretical formulations.

4 “Rare disease” is merely a statistical classification (see Huyard 2009 on the emergence of this concept); within the European Union, a rare disease is defined as one by which no more than 5 in 10,000 persons are affected. The term ‘‘rare’’ should, however, not hide the fact that, with an estimated number of 5,000–8,000 rare diseases, about 30 million people are affected only in the EU.

5 It is misleading, therefore, when Bogner (2012) contrasts “laboratory participation” with “protest participation” since “uninvited” civil society involvement is by no means limited to protest, although the latter is doubtlessly a both important and legitimate form of public participation in science and technology.

6 While I adopt the distinction of activist vs. deliberative participation from Young (2001), I use these terms in the context of science and technology in a slightly different manner than Young.

7 This model of participation does of course not in the same manner apply to the activities of environmental or consumer organizations, since the engagement of the latter frequently is not equally driven by the immediate needs of specific social groups but rather oriented towards more general social and political aims. One should, however, not fail to see that in many cases environmental organizations or social movements also act as a kind of interest groups, for instance when they protest against local or regional environmental pollutions which may have serious public health consequences. Thus, there is no clear-cut distinction between interest groups and CSOs with broader goals. The so-called “Environmental Breast Cancer Movement” to which I will refer below is an illuminating example of how group-related needs and more general political concerns interact.

8 However, the question of success of civil society involvement in research and technology is far more complex than is usually assumed. I cannot elaborate on this here; for an illuminating discussion with regard to patient associations see Epstein (2011).

9 However, there is more to the issue of representation than this (see Brown 2006), because even in small interest groups (such as are many patient associations) some members usually are represented by others (Epstein 2011).

10 See with regard to patient organizations Tomes and Hoffman (2011).

11 The “Dominant Epidemiological Paradigm” (DEP) of cancer research nicely illustrates how such internal and external influences interact and reinforce each other (see Brown 2007).

12 With his notion of “well-ordered science” Philip Kitcher (2001, 2011) has proposed a different solution to the problems related to research agenda setting. Following Kitcher, a group of well-informed deliberators should rationally decide what research topics should be selected and how they should be approached by science. While this proposal might indeed lead to practices which are more accountable than the current dynamics of research agenda setting, it raises itself a number of difficult problems. Besides the above-mentioned general problems of deliberative processes (e.g., the factual exclusion or self-exclusion of certain actors and styles of reasoning), the questions will arise of how, by whom and on what grounds those deliberators should be elected. .

13 The distinction is nevertheless crucial in order to challenge the equation of public participation with participating in invited pTA exercises. Yet the invited vs. uninvited distinction becomes questionable in itself when it is essentialized in the sense that uninvited participation is generally held to be “better” than invited one (or vice versa) and thus distracts the analytical attention from asking *how* they are respectively shaped.

14 This deliberate open-endedness might be able to counteract the “primacy of organization” criticized by Bogner and others. Participatory processes should not simply be seen as a failure if they do not produce the expected standard outcomes.

15 In 2008 the group changed its name to Nanotechnology Citizen Engagement Organization (NanoCEO); since then it is active in the field of nanotechnology politics. See http://www.nanoceo.net/about (last access 8.6.2012).

16 As Hess (2011: 639) rightly remarks, such procedures would, however, most likely be jeopardized by attempts at manipulation and capture by powerful interested parties.
